# Effect of Nanohydroxyapatite and Silver Nanoparticle Incorporation on the Flexural Strength of Resin Composites

**DOI:** 10.1155/bmri/9132555

**Published:** 2025-08-27

**Authors:** Marzie Moradaian, Maryam Saadat, Shahab Agharezaei, Zahra Khorshidi Asl

**Affiliations:** ^1^ Department of Operative Dentistry, Shiraz University of Medical Sciences, Shiraz, Fars Province, Iran, sums.ac.ir; ^2^ Student Research Committee, Department of Operative Dentistry, Shiraz University of Medical Sciences, Shiraz, Fars Province, Iran, sums.ac.ir

**Keywords:** composite resin, flexural strength, nanohydroxyapatite, silver nanoparticles

## Abstract

This in vitro study investigated the impact of silver nanoparticles (AgNPs) and nanohydroxyapatite (nanoHAP) on the flexural strength of dental composite resin. Fifty composite samples were prepared in five groups (*n* = 10): (1) nonspatulated composite resin (negative control); (2) hand‐spatulated composite resin (positive control); (3) composite resin and 2 wt% nanoHAP; (4) composite resin and 1 wt% AgNPs; and (5) composite resin, 2 wt% nanoHAP, and 1 wt% AgNPs. Flexural strength was measured using a universal testing machine. Scanning electron microscopy (SEM) was used to evaluate the dispersion of nanoparticles in the composite resin. The data were analyzed using the Kruskal–Wallis and Mann–Whitney tests (*p* < 0.05 was considered significant). The highest mean flexural strength was observed in the negative control, while the lowest flexural strength was seen in Group 4 (composite resin and AgNPs). The nanoHAP group demonstrated significantly higher flexural strength than the AgNP group (*p* = 0.043). However, no significant difference was observed between the nanoHAP‐only group and the group containing both nanoHAP and AgNPs (*p* = 0.075). These findings suggest that nanoHAP‐loaded composite resin possesses higher flexural strength than AgNP‐incorporated composite resin. Additionally, comparisons with the positive control group indicated that the addition of nanoparticles did not necessarily result in significant improvement in flexural strength. These findings can pave the way for future studies on optimizing the composition of advanced dental materials with enhanced longevity and improved mechanical properties.

## 1. Introduction

Composite resins are extensively used in modern dentistry for cosmetic and restorative applications. They play a crucial role in restoring both tooth function and aesthetics. To improve clinical outcomes, manufacturers aim to enhance the mechanical strength and antimicrobial properties of these materials, thereby improving their resistance to masticatory forces and minimizing the risk of secondary caries [1–4]. Despite significant advancements in the field, challenges persist in achieving optimal toughness, strength, and longevity of composite restorations in the high‐stress oral environment [5, 6].

Nanotechnology has emerged as a promising avenue in dental material research, offering novel solutions to improve composite resin performance [7]. Nanoparticles—ultrafine particles with dimensions less than 100 nm—have become integral to a range of dental applications [8].

One such nanoparticle is nanohydroxyapatite (nanoHAP), an inorganic compound naturally found in hard tooth tissue, bones, and pathologically calcified tissues [9–11]. Synthetic HAP offers distinct advantages due to its chemical similarity to natural hydroxyapatite. This synthetic material is biocompatible, bioactive, and nontoxic, showing no inflammatory or carcinogenic effects [12–15]. The addition of HAP to composite materials has been shown to influence their mechanical properties significantly [16–18].

Silver nanoparticles (AgNPs) are also widely studied for their antimicrobial properties. They have been shown to combat plaque formation, bacterial growth, and fungal infections in the oral cavity [19]. Additionally, some studies suggest that AgNPs may enhance mechanical properties such as strength and elasticity without compromising biocompatibility [20–23]. However, the evidence regarding their effects on composite resin strength is limited and inconsistent [19, 24].

Flexural strength is a key parameter in assessing the mechanical performance of composite resins. It represents the material’s ability to resist bending forces without fracture and is commonly measured using the three‐point bending test [25, 26]. While both AgNPs and nanoHAP have been independently studied for their impact on composite properties, no previous research has evaluated the effects of the simultaneous incorporation of these two materials on the bending properties of dental resin composites. Therefore, this study was aimed at investigating the individual and combined incorporation of AgNPs and nanoHAP on the flexural strength of dental resin composite.

## 2. Materials and Methods

In this study, 50 bar‐shaped composite resin specimens (Filtek Z250 composite resin, 3M ESPE, St. Paul, Minnesota, United States) were prepared and divided into five groups (*n* = 10). The sample size was calculated using the *F*‐test family for a one‐way ANOVA involving five resin composite groups. With 10 experimental units per group, a minimum detectable effect size of *f* = 0.51 could be achieved, corresponding to a statistical power of 80% (*β* = 0.20) at a significance level of 5% (*α* = 0.05). Group 1 was a nonspatulated composite resin (negative control). The second group was a hand‐spatulated composite resin (positive control). In this group, uncured Z250 composite resin was hand‐spatulated with a plastic instrument for 30 min in a dark environment. In Group 3, the composite resin was mixed into nanoHAPs (Sigma‐Aldrich Inc., St. Louis, Missouri, United States) with a grain size of < 200 nm. The nanoparticles were manually incorporated into uncured Z250 resin at a concentration of 2% by weight and mixed for 30 min under light‐protected conditions to achieve a homogeneous and workable material consistency. However, it is important to acknowledge that manual mixing may introduce voids and inhomogeneities, potentially compromising the mechanical performance of the final composite [27]. The operator’s technique plays a critical role in this process, as the irregular formation of air bubbles during mixing can adversely affect both the structural integrity and the reliability of subsequent test results. The mixing procedure was performed on a glass slab over a wide area and under the safelight condition to avoid interference from surrounding light. In the fourth group, AgNPs (USA Research, Nanomaterial Inc., United States) with an average size of > 20 nm (USA Research, Nanomaterial Inc., United States) were incorporated into the composite resin at 1 wt% using the same manual mixing procedure as described for the previous group. In Group 5, nanoHAP and AgNPs were manually incorporated into the composite resin at 2 and 1 wt%, respectively, as described for Groups 3 and 4.

All specimens were prepared in a metal, rectangular‐shaped mold with a central fissure. The size of this fissure in each mold was 2 × 2 × 25 mm to match the standards according to ISO 4049/2009 standards. The molds were placed on a glass slab covered with a polyester film strip (Mylar, DuPont Teijin Films, Wilmington, Delaware, United States), and composite resin was inserted into the molds. A second polyester film strip and a glass slab were then placed on the top of the molds to remove the excess material.

To ensure optimal polymerization, the samples were initially light‐cured at four distinct locations using an LED curing unit (Demetron, Kerr Corporation, Orange, California, United States) with an intensity of 1200 mW/cm^2^, with each spot irradiated for 20 s. Following removal from the molds, the specimens were further light‐cured at four corresponding locations on the opposite side, with each point again being exposed to the curing light for 20 s to ensure complete curing. Subsequently, all samples were immersed in distilled water at room temperature for 24 h before conducting the three‐point flexural strength test.

Scanning electron microscopy (SEM) (Model LEO 1400, England) was used to evaluate the dispersion of nanoparticles within the resin matrix for Groups 3, 4, and 5. Before analysis, all samples were sputter‐coated with a thin layer of gold. Images were taken at two magnifications: ×2850 and ×14,000.

The flexural strength of the samples was measured using a universal testing machine (ZWICK/ROELLZ020, ULM, Germany) with a crosshead speed of 0.5 mm/min, and the maximum load was recorded before fracture. Flexural strength was calculated using Equation (1), where *S* is the flexural strength (megapascals) and *F* and *L*, respectively, are the maximum applied force to the samples and the span between two supports (20 mm). *B* is the width, and *H* refers to the height of the specimens.

(1) S=3FL2BH2.

Statistical analysis was performed using SPSS software (Version 17, IBM SPSS, Chicago, Illinois, United States). Nonparametric Kruskal–Wallis test and multiple Mann–Whitney tests were used to assess significant differences between five groups of specimens. Differences between the groups were considered statistically significant with *p* < 0.05.

## 3. Results

The average, minimum, maximum, and standard deviations of flexural strength values for all groups are presented in Table 1. The highest mean flexural strength was observed in the first group of specimens (negative control) with a value of 101.81 MPa. The lowest flexural strength was related to Group 4 (AgNP‐containing methacrylate composite resins) with a value of 69.94 MPa. The Kruskal–Wallis test results indicated a statistically significant difference (*p* < 0.001) among the methacrylate composite resins reinforced with different nanoparticles (Groups 3, 4, and 5).

**Table 1:** Flexural strength of different groups of specimens. Group 1, composite resin; Group 2, spatulated composite resin; Group 3, nanoHAP and composite resin; Group 4, AgNP and composite resin; and Group 5, nanoHAP, AgNP, and composite resin.

**Groups**	**Mean**	**SD**	**Minimum**	**Maximum**
1	101.81	5.78282	90.80	109.00
2	72.18	7.75970	57.60	81.90
3	80.36	11.5706	61.40	93.90
4	69.94	7.17576	61.30	84.10
5	70.52	11.01452	52.30	83.50
Total	78.96	14.86644	52.30	109.00

According to the results of the Mann–Whitney test, the negative control group exhibited a statistically significantly higher flexural strength compared to Groups 2, 3, 4, and 5 (*p* < 0.001). In contrast, no statistically significant difference was observed between the mean flexural strength of the positive control group and that of the nanoparticle‐reinforced composite resin groups (*p* > 0.05).

Specimens in Group 3 (nanoHAP and composite resin) displayed a significant difference (*p* = 0.043) in mean flexural strength compared to Group 4 (AgNPs and composite resin). No statistically significant differences were found between Groups 3 and 5 (*p* = 0.075) or between Groups 4 and 5 (*p* = 0.971). A summary of *p* values is presented in Table 2.

**Table 2:** Summary of *p* values of the Mann–Whitney test. Group 1, composite resin; Group 2, spatulated composite resin; Group 3, nanoHAP and composite resin; Group 4, AgNP and composite resin; and Group 5, nanoHAP, AgNP, and composite resin.

**Groups**	**1**	**2**	**3**	**4**	**5**
1	—	0.000 ^∗^	0.000 ^∗^	0.000 ^∗^	0.000 ^∗^
2	0.000 ^∗^	—	0.082	0.257	0.940
3	0.000 ^∗^	0.082	—	0.043 ^∗^	0.075
4	0.000 ^∗^	0.257	0.043 ^∗^	—	0.971
5	0.000 ^∗^	0.940	0.075	0.971	—

^∗^Statistically significant at *P* value < 0.05.

**Figure 1:**
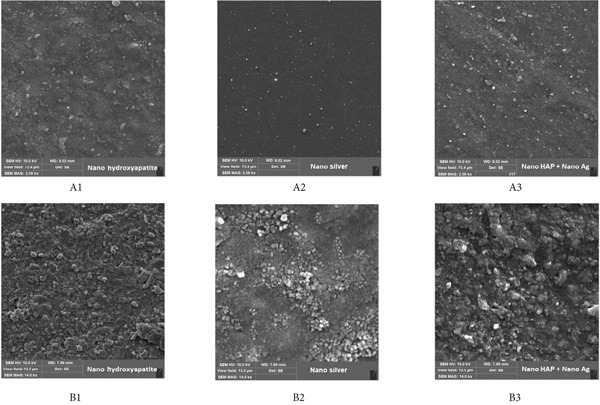
Surface morphologies of the samples obtained from SEM of Groups 3, 4, and 5 at two different magnifications of ×2580 (A1, A2, and A3) and ×14000 (B1, B2, and B3) A1 and B1, nanoHAP and composite resin with relatively uniform dispersion and minimal clustering; A2 and B2, AgNP and composite resin with more aggregated dispersion and more clusters; A3 and B3, nanoHAP, AgNP, and composite resin with more heterogeneous dispersion of nanoparticles and partial clustering of both nanoparticles.

The results of SEM images of randomly selected specimens of Groups 3, 4, and 5 at two different magnifications of × 2580 (A1, A2, and A3) and ×14,000 (B1, B2, and B3) have been represented in Figure 1. In Group 3 (nanoHAP + resin composite), SEM images revealed a relatively uniform dispersion of nanoHAP particles within the resin matrix, with minimal clustering. In Group 4 (AgNP + resin composite), AgNPs appeared more aggregated, showing clusters that may have contributed to stress concentration points, potentially explaining the lower flexural strength. In Group 5 (nanoHAP + AgNP + resin composite), partial clustering of both nanoparticles was observed, but the distribution was more heterogeneous compared to Groups 3 and 4.

## 4. Discussion

This study evaluated the impact of incorporating nanoHAP and AgNPs on the flexural strength of the resin composite. The results showed that nanoHAP‐incorporated composites exhibited improved flexural strength compared to the AgNP group (*p* = 0.043). However, nanoparticle incorporation did not lead to statistically significant improvements in flexural strength compared to the positive control group. Moreover, the observed changes in flexural strength across groups may not translate to clinically meaningful advantages. Their impact on durability or clinical restorative performance remains uncertain. Therefore, long‐term in vivo evaluations are necessary to verify the relevance of these findings.

Flexural strength is a key mechanical property in dental restorations, as it determines a material’s ability to resist masticatory forces and complex stress [28, 29]. Recent efforts have focused on improving dental composite resins and optimizing their filler particles. Numerous studies have explored the incorporation of nanoparticles as filler components to promote the clinical performance of resin composites. NanoHAP particles are among the frequently used nanoparticles because of their great biocompatibility and appropriate color. AgNPs have also been employed as composite nanofillers and have desirable antimicrobial and biological properties [30–34].

In the present study, the composite reinforced with nanoHAP demonstrated higher flexural strength compared to the one reinforced with AgNPs. This variation in flexural strength between the two types of reinforcements may be attributed to differences in their dispersion within the resin matrix. Previous research has shown that uniform dispersion of nanofillers in the resin matrix improves the mechanical strength of the resin composites [35, 36]. In contrast, nanofillers without surface treatment tend to agglomerate, acting as stress initiation sites and thereby compromising the composite’s strength. In this study, since the nanofillers were not surface treated, the SEM analysis revealed that nanoHAP was better dispersed, whereas AgNPs showed noticeable clustering. This difference in dispersion could explain the reduced flexural strength observed with AgNPs reinforcement compared to nanoHAP. Similar to the present study, Alla et al. also reported that AgNPs tended to agglomerate as their concentration increased in the resin matrix and decreased the flexural strength of resin‐based materials [37].

The reduced flexural strength observed in the hand‐spatulated group (positive control), compared to the nonspatulated control, aligns with previous findings, suggesting that manual mixing introduces voids that impair mechanical performance [27]. Therefore, operators should be aware that the mixing method can significantly influence outcomes, as heterogeneous bubble formation may adversely affect test results.

It has been established in previous studies that incorporating nanoHAP into dental composites can positively impact mechanical properties, especially the flexural strength of the composite resin [2]. Incorporation of higher percentages of nanoHAP to a polymer material makes it less permeable to the blue light emitted by LED lights, while using lower percentages of nanoHAP results in a better curing depth [38]. Moharam et al. investigated the effects of four different percentages of nanoHAP (0, 1.5, 2, and 5 wt%) on flexural strength. They concluded that utilizing 2 wt% of nanoparticles could increase the flexural strength of the material, and flexural strength decreased for higher weight percentages [2]. Therefore, in the present study, 2 wt% nanoHAP was incorporated into the composite resins. The results indicate that nanoHAP‐containing samples exhibited lower flexural strength than the negative control group. Although the nanoHAP‐reinforced composite showed an 11% increase in flexural strength compared to the positive control group, this increase was statistically insignificant.

AgNPs were also mixed into the dental composite in the present study (Group 4). AgNPs have been blended with various materials in dentistry to utilize their antimicrobial characteristics, which disrupt bacteria and improve oral health [19]. According to Liu et al., incorporating 50 ppm silver nanocrystals into the composite resin resulted in improved mechanical properties and an antimicrobial effect. However, the mechanical properties and color of the composite resin were negatively impacted when more than 50 ppm of AgNPs were added to the material [32].

Based on the results by Kasraei and Azarsina, adding silver particles to Z250 and P90 composites at 0.05 or 0.1 wt% showed that low percentages of AgNPs did not affect mechanical properties, but they were beneficial to enhance the antimicrobial effects of the composite [39]. The present study reported that reinforcing composites with 1 wt% of AgNPs resulted in a 3.6% decrease in flexural strength compared to that of the positive control group (spatulated composite); also, the lowest flexural strength was related to AgNP‐reinforced composites. However, the difference between the positive control group and the AgNP‐containing group was not statistically significant (*p* = 0.257).

Recent advances in biomaterial research have highlighted the role of silver‐doped HAP in enhancing the mechanical properties and antibacterial efficacy of dental composites. Studies have demonstrated that silver‐doped HAP improves compressive strength and bonding adhesion, with an observed 5.52% increase in compressive strength at 0.2 wt% silver doping [40]. Additionally, silver incorporation has been shown to reduce porosity, achieving a minimum of 7.969%, which can contribute to improved structural integrity [40]. In the context of tribological behavior, silver‐doped HAP reinforcement in PMMA‐based composites exhibits a progressive increase in hardness and a reduction in wear rate, particularly at higher reinforcement levels [41]. This enhanced durability is further supported by improved hydrophilicity and cohesion strength, making such biocomposites highly relevant for dental applications. Furthermore, investigations into AgNP adhesion on HAP surfaces suggest that increasing AgNO_3_ concentrations leads to greater bioactivity and apatite formation, which is particularly beneficial for biomedical applications requiring antibacterial properties [42]. Given these findings, incorporating nanoHAP and AgNPs into resin composites holds promise for enhancing flexural strength, durability, and antimicrobial performance, making them a viable material choice for modern dental restorative applications.

In this study, the combination group (nanoHAP + AgNPs) did not show significant improvements, with a 2.8% decrease in flexural strength compared to the positive control. Various factors may account for this slight decrease in the flexural strength of Groups 4 and 5 (AgNP‐containing group and nanoHAP AgNP‐containing group). As mentioned, the manual mixing method used in this study may result in heterogeneous void formation, which leads to lower flexural strength. During the curing process of AgNP‐containing composites, we noticed that after 20 s of curing from above, the bottom layer of the AgNP‐containing composite remained relatively soft after removing samples from the mold. Therefore, it was concluded that AgNPs might reduce light transmission into deeper parts of composites. AgNP‐containing samples are more opaque and have less curing depth, which could be a convincing explanation for their weaker mechanical properties and lower flexural strength. Moreover, it was investigated in a previous study by Kattan et al. that silver‐containing composites exhibit lower curing depth compared to the control group [43]. However, further studies are required to clarify the impacts of using different nanoparticles in dental composite resins. Clinicians should be cautious when considering nanoparticle‐modified composites, as benefits in flexural strength are not guaranteed. Importantly, the antimicrobial potential of AgNPs may offer clinical value but should be balanced against potential compromises in mechanical properties. Future studies should explore how these materials perform under functional loading and long‐term in vivo conditions.

Our findings partially support previous studies. For example, Moharam et al. [2] found that incorporating 2 wt% nanoHAP significantly improved the flexural strength of composite resins, which is in agreement with the trend observed in our study. Similarly, Kasraei et al. showed that low concentrations of AgNPs (0.05–0.1 wt%) did not adversely affect mechanical strength while enhancing antimicrobial effects, partially supporting our results in Group 4 [1].

However, other studies contradict our findings. Liu et al. demonstrated a significant improvement in mechanical strength after incorporating 50 ppm AgNPs [32], while in our study, 1 wt% AgNP led to a slight (3.6%) decrease in flexural strength. Murugan et al. also reported mixed outcomes regarding the mechanical performance of HAP/AgNP composites, highlighting how formulation differences can yield conflicting results [44]. However, further studies are required to clarify the impacts of using different nanoparticles in dental composite resins. In a recent publication, Haque et al. developed nanoHAP‐based composite scaffolds for maxillofacial reconstruction and emphasized their potential in clinical applications. They also highlighted the need for in vivo mechanical validation [45].

The mechanical performance of resin composites is influenced not only by the type and concentration of nanofillers but also by their particle size and morphology. Although the present study did not vary the size or shape of the incorporated AgNPs and nanoHAP, previous investigations have demonstrated that smaller nanoparticles, due to their higher surface area‐to‐volume ratio, may enhance interfacial bonding and stress distribution within the resin matrix [46]. Excessively small particles can agglomerate, potentially compromising mechanical integrity [47]. However, variations in nanoparticle dimensions may not always translate into significant mechanical differences. For instance, a previous investigation assessing the inclusion of AgNPs of multiple sizes into irreversible hydrocolloid reported no notable change in gel strength across 0.5 and 1.0 wt% concentrations, regardless of particle size range [48]. Similarly, the shape of nanofillers—whether spherical, rod‐like, or plate‐like—can affect load transfer efficiency and filler dispersion [46]. In our formulation, commercially available AgNPs (~20 nm) and nanoHAP (< 200 nm) were selected based on prior evidence supporting their biocompatibility and mechanical stability at these dimensions [33]. While the current study maintained consistent particle characteristics, future work may explore how tailored size and shape modifications influence flexural strength outcomes.

Environmental conditions such as temperature and humidity were not strictly controlled during the 3‐point bending test. This may have influenced the flexural strength results and should be considered a limitation. Additionally, the manual mixing technique used for nanoparticle incorporation may introduce variability and hinder reproducibility due to nonhomogeneous dispersion. Although economic limitations necessitated this method, future studies should adopt standardized mechanical mixing techniques for greater homogeneity and scientific rigor. To maximize polymerization and enhance the mechanical properties of nanoparticle‐reinforced composite resin, utilizing light‐transmitting molds for deeper light penetration and incorporating a coupling agent can be highly effective. Although SEM imaging confirmed the presence of nanoparticles within the resin matrix, the study did not assess the quantitative rate of nanoparticle dispersion. This parameter may critically influence the degree of matrix‐filler interaction, curing depth, and overall mechanical behavior, suggesting further investigation using energy‐dispersive x‐ray spectroscopy (EDX) or transmission electron microscopy (TEM) for detailed analysis.

## 5. Conclusion

Within the limitations of this study, nanoHAP‐incorporated composite resin demonstrated higher flexural strength than AgNP‐containing composites. However, the flexural strength did not show a statistically significant improvement when compared to the positive control group. These findings suggest that adding nanoHAP or AgNPs did not significantly enhance the flexural strength of the tested composite resin under the specified conditions.

## Conflicts of Interest

The authors declare no conflicts of interest.

## Funding

No funding was received for this manuscript.

## Data Availability

The data used in the current study are available from the corresponding author upon reasonable request.
